# Leukopenia and leukocytosis are associated with early postoperative complications following aseptic revision total shoulder arthroplasty

**DOI:** 10.1007/s00590-025-04343-z

**Published:** 2025-06-02

**Authors:** Steven H. Liu, Rustin Mahboubi Ardakani, Rachel A. Loyst, Allen Bramian, Edward D. Wang

**Affiliations:** 1 University of Southern California, Los Angeles, USA; 2 Stony Brook Medicine, Stony Brook, USA

**Keywords:** Revision total shoulder arthroplasty, Total shoulder arthroplasty, White blood cells, Leukocytosis, Leukopenia, Complications

## Abstract

**Background:**

This study investigates the association between preoperative leukopenia and leukocytosis with 30-day postoperative complications following noninfectious revision total shoulder arthroplasty (TSA).

**Methods:**

The American College of Surgeons National Surgical Quality Improvement Program database was queried for all patients who underwent noninfectious revision TSA from 2015 to 2022. The study population was divided into three groups based on preoperative white blood cell (WBC) count: normal (WBC 4500–11,000), leukopenia (WBC ≤ 4500), and leukocytosis (WBC ≥ 11,000). Logistic regression analysis was conducted to investigate the relationship between WBC count and postoperative complications.

**Results:**

Compared to normal WBC counts, leukocytosis was independently associated with an increased likelihood of experiencing any complication (OR 1.71, 95% CI 1.13–2.59; *P* = 0.012), sepsis (OR 5.31, 95% CI 1.38–20.37; *P* = 0.015), non-home discharge (OR 2.18, 95% CI 1.18–4.05; *P* = 0.013), readmission (OR 2.76, 95% CI 1.36–5.63; *P* = 0.005), and LOS > 2 days (OR 1.68, 95% CI 1.06–2.66; *P* = 0.028). Compared to normal WBC counts, leukopenia was independently associated with an increased likelihood of experiencing pneumonia (OR 14.98, 95% CI 2.32–96.56; *P* = 0.004) and readmission (OR 2.78, 95% CI 1.49–5.17; *P* = 0.001).

**Conclusion:**

The present study identified preoperative leukocytosis and leukopenia as independent risk factors for 30-day postoperative complications following revision TSA. Integrating WBC count into preoperative assessments can enhance the identification of patients at risk for postoperative complications, allowing for more tailored management strategies and potentially improving overall patient outcomes.

## Introduction

Total shoulder arthroplasty (TSA) is a common and effective procedure for treating osteoarthritis, rotator cuff damage, and fracture. As the need for TSA rises alongside an aging population, the risk of complication and subsequent need for revision surgery rises [1, 2]. Compared to primary TSA, revision TSA has been associated with higher complication rates [3, 4]. Therefore, preoperative risk stratification using available data such as preoperative laboratory values may help surgeons better select surgical candidates and improve patient outcomes after aseptic revision TSA.

Common preoperative assessments often involve blood work including complete blood count (CBC), metabolic panels, and coagulation studies. Using easily obtainable lab data can be a helpful tool in predicting postoperative complications. Previous research has highlighted preoperative hematocrit, international normalized ratio (INR) and blood urea nitrogen (BUN) as informative laboratory studies that can help assess a patient’s preoperative risk [5–8]. One potentially useful component of a routine CBC is the white blood cell (WBC) count. White blood cells (leukocytes) are essential to immune defense and provide a window into a patient’s inflammatory status and ability to fight infection [9, 10].

Prior studies have identified leukopenia as an independent predictor of postoperative complications following TSA and leukocytosis as an independent predictor of complications following both TSA and revision TSA [11, 12]. Other studies have examined postoperative leukocyte counts after total hip and knee arthroplasty—primarily as a diagnostic marker to guide the evaluation of postoperative infection—but did not evaluate preoperative leukocyte levels for risk stratification prior to surgery. [13, 14].

In this study, we investigate preoperative leukocyte counts and postoperative complications following revision TSA. In patients undergoing aseptic revision total shoulder arthroplasty, we hypothesize that both preoperative leukopenia (≤ 4500 cells/µL) and leukocytosis (≥ 11,000 cells/µL) will be independently associated with higher odds of postoperative complications—specifically infectious events (sepsis, pneumonia, SSI), prolonged length of stay, non-home discharge, unplanned reoperation, and 30-day readmission—compared to patients with normal preoperative WBC counts. As revision TSA becomes more common, harnessing preoperative risk-assessment tools has the potential to markedly improve outcomes, especially in patients with substantial comorbidity burdens.

## Materials and methods

The American College of Surgeons National Surgical Quality Improvement Program (ACS-NSQIP) database was queried for all patients who underwent revision TSA from 2015 to 2022. This study was exempt from approval by our University’s Institutional Review Board because the NSQIP database is fully deidentified. Data in the NSQIP database is gathered from over 600 hospitals in the United States by trained surgical clinical reviewers. The data is periodically reviewed to maintain high reliability.

**Fig. 1:**
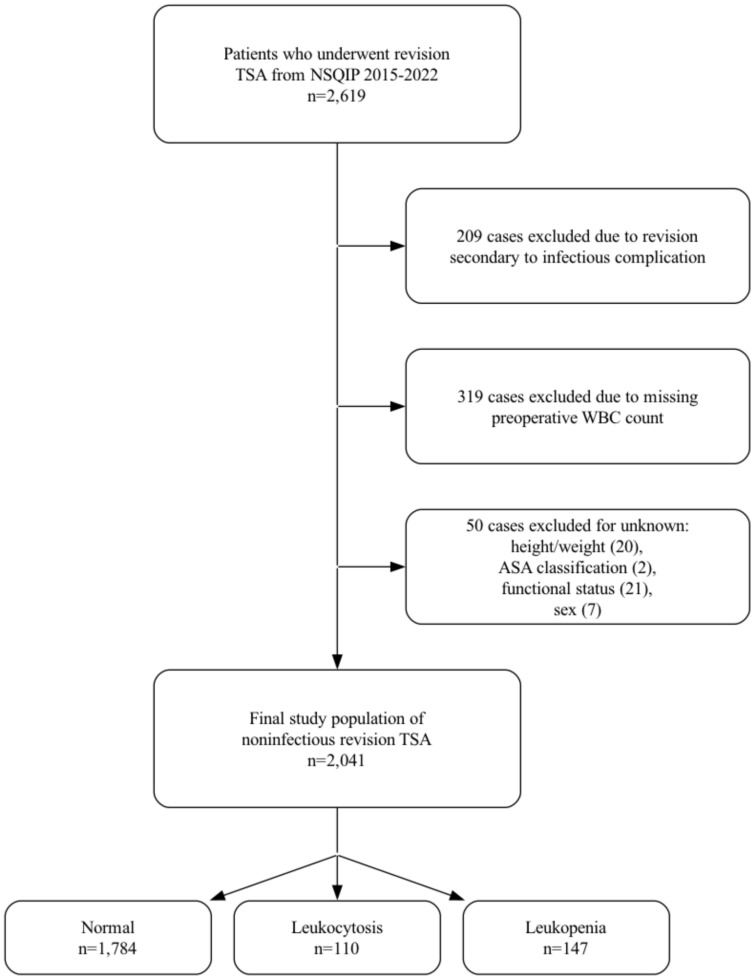
Case selection schematic. TSA, total shoulder arthroplasty; *NSQIP*, National Surgical Quality Improvement Program; WBC, white blood cell; ASA, American Society of Anesthesiologists

The *Current Procedural Terminology* (CPT) codes 23473 and 23474 were used to identify 2619 patients who underwent revision TSA from 2015 to 2022 (Fig. 1). The NSQIP database inherently excludes all cases for patients younger than 18 years of age and all cases with primary admission related to trauma. First, 209 revision TSA cases were excluded due to revision for an infectious etiology because the NSQIP database does not contain details regarding the nature of the infection (e.g., acute vs. chronic), which may impact complication rates. Next, 319 cases were excluded due to missing preoperative WBC counts. Additionally, 50 cases were excluded for unknown height/weight, American Society of Anesthesiologists (ASA) classification, functional health status, or sex. A total of 578 cases were excluded from the initial cohort: 209 revision TSAs performed for infectious etiologies (due to lack of detail on infection chronicity), 319 with missing preoperative WBC values, and 50 with incomplete demographic or clinical data (unknown height/weight, ASA classification, functional health status, or sex). The final cohort included 2,041 patients, stratified by preoperative WBC into three groups: normal (4500–11,000 cells/µL; *n* = 1784), leukocytosis (≥ 11,000 cells/µL; n = 110), and leukopenia (≤ 4500 cells/µL; *n* = 147). These values are based on standardized thresholds from previously validated studies [12, 15].

Variables collected in this study included patient demographics, comorbidities, surgical characteristics, and 30-day postoperative complication data. Patient demographics included sex, age, body mass index (BMI), functional status, ASA classification, smoking status, and preoperative steroid use. Preoperative comorbidities included congestive heart failure (CHF), hypertension, severe chronic obstructive pulmonary disease (COPD), bleeding disorders, disseminated cancer, and total operation time. Thirty-day complications included the following: sepsis, septic shock, pneumonia, unplanned reintubation, urinary tract infection (UTI), cardiac arrest or myocardial infarction (MI), stroke, blood transfusions, deep vein thrombosis (DVT), pulmonary embolism (PE), on ventilator > 48 h, surgical space infection (SSI), wound dehiscence, acute renal failure, *Clostridioides difficile*** (***C. diff*) infection, non-home discharge, readmission, unplanned reoperation, periprosthetic fracture, length of stay (LOS) > 2 days, and mortality. We defined prolonged length of stay as > 2 days based on established precedent in orthopedic literature, where the median LOS for elective joint and spine procedures is typically 1–2 days. This cutoff corresponds to the upper quartile of expected recovery in high-volume centers and has been shown to identify patients at increased risk for postoperative complications and higher resource utilization.

Statistical analyses were performed using Python version 3.8 with the Statsmodels Python package. Univariate analysis was used to compare patient demographics and comorbidities between the two groups. Multivariable logistic regression—adjusted for significant and near-significant demographic factors and comorbidities associated with WBC status—was used to evaluate the independent effects of leukopenia and leukocytosis on postoperative complications. Odds ratios (OR) were reported with 95% confidence intervals (CI). The level of statistical significance was set at *P* < 0.05.

## Results

A total of 2,041 patients met inclusion criteria and were categorized by preoperative WBC: 1784 (87.4%) with normal counts (4500–11,000 cells/µL, mean 7190, SD 1580), 110 (5.4%) with leukocytosis (≥ 11,000 cells/µL, mean 12,880, SD 3340), and 147 (7.2%) with leukopenia (≤ 4500 cells/µL, mean 3910, SD 490) (Table 1, Table 2). This group of patients was 52.4% female, had a mean age of 68.2, mean BMI of 31.3, 96.4% were of independent functional status, 35.7% were of ASA classification ≤ 2, 10.8% were active smokers, 6.1% were chronic steroid users, 1.7% had CHF, 67.0% had hypertension, 7.6% had COPD, 3.6% had a bleeding disorder, 0.3% had disseminated cancer, and had a mean hospital LOS of 1.8 days (Table 1).

**Table 1:** Demographics and comorbidities of overall patient cohort

Characteristics	Number (%)/Mean ± SD
Overall	2041 (100.0)
Sex	
Female	1069 (52.4)
Male	972 (47.6)
Age	68.2 ± 9.7
BMI (kg/m^2)	31.3 ± 6.9
Functional status prior to surgery	
Dependent	74 (3.6)
Independent	1967 (96.4)
ASA classification	
≤ 2	729 (35.7)
≥ 3	1312 (64.3)
Smoker	
No	1820 (89.2)
Yes	221 (10.8)
Steroid use	
No	1916 (93.9)
Yes	125 (6.1)
Comorbidities	
CHF	35 (1.7)
Hypertension	1368 (67.0)
COPD	155 (7.6)
Bleeding disorder	73 (3.6)
Disseminated cancer	6 (0.3)
LOS	1.8 ± 2.4

*BMI* body mass index; *ASA* American Society of Anesthesiologists; *CHF* congestive heart failure; *COPD* chronic obstructive pulmonary disease; *LOS* length of stay

After univariate analysis, leukocytosis—versus normal WBC—was significantly associated with higher ASA class (≥ 3; *P* = 0.010), active smoking (*P* = 0.003), and chronic steroid use (*P* = 0.034) (Table 2). It was also linked to greater rates of any complication (*P* = 0.018), sepsis (*P* = 0.011), surgical site infection (*P* = 0.040), non-home discharge (*P* = 0.026), readmission (*P* = 0.007), unplanned reoperation (*P* = 0.044), and length of stay > 2 days (*P* = 0.041) (Table 3). Multivariable logistic regression, adjusted for all significant (*P* < 0.05) and near-significant (*P* < 0.2) patient demographics and comorbidities, revealed that patients with baseline leukocytosis undergoing aseptic revision TSA had significantly higher adjusted odds of any complication (OR 1.71, 95% CI 1.13–2.59; *P* = 0.012), sepsis (OR 5.31, 95% CI 1.38–20.37; *P* = 0.015), non-home discharge (OR 2.18, 95% CI 1.18–4.05; *P* = 0.013), readmission (OR 2.76, 95% CI 1.36–5.63; *P* = 0.005), and LOS > 2 days (OR 1.68, 95% CI 1.06–2.66; *P* = 0.028) compared to those with baseline normal WBC counts (Table 4). Of the patients discharged to non-home locations from the normal cohort, 62 (50%) patients were discharged to skilled care, 24 (19.4%) to rehab, 7 (5.6%) to acute care, and 31 (25.0%) to an other facility. Of the patients discharged to non-home locations from the leukocytosis cohort, 11 (78.6%) were discharged to skilled care, 1 (7.1%) to rehab, 0 (0%) to acute care, and 2 (14.3%) to an other facility (Table 5).

**Table 2:** Demographics and comorbidities of patients with normal WBC count, leukocytosis, and leukopenia. Bold *P* values indicate statistical significance with *P* < .05

Characteristics	Normal (4500–11,000)	Leukocytosis (≥ 11,000)		Leukopenia (≤ 4500)	
	Number (%)/ Mean ± SD	Number (%)/ Mean ± SD	*P* value	Number (%)/ Mean ± SD	*P* value
WBC count	7.19 ± 1.58	12.88 ± 3.34		3.91 ± 0.49	
Overall	1784 (100.0)	110 (100.0)		147 (100.0)	
Sex			0.285		0.808
Female	928 (52.0)	63 (57.3)		78 (53.1)	
Male	856 (48.0)	47 (42.7)		69 (46.9)	
Age			0.247		0.088
18–39	14 (0.8)	2 (1.8)		2 (1.4)	
40–59	279 (15.6)	20 (18.2)		29 (19.7)	
60–79	1,283 (71.9)	77 (70.0)		103 (70.1)	
≥ 80	208 (11.7)	11 (10.0)		13 (8.8)	
BMI (kg/m^2)			0.096		**0.001**
< 18.5	5 (0.3)	0 (0.0)		5 (3.4)	
18.5–24.9	286 (16.0)	16 (14.5)		33 (22.4)	
25–29.9	542 (30.4)	24 (21.8)		44 (29.9)	
≥ 30	951 (53.3)	70 (63.6)		65 (44.2)	
Functional status prior to surgery			0.651		0.305
Dependent	66 (3.7)	5 (4.5)		3 (2.0)	
Independent	1,718 (96.3)	105 (95.5)		144 (98.0)	
ASA classification			**0.010**		0.131
≤ 2	641 (35.9)	26 (23.6)		62 (42.2)	
≥ 3	1143 (64.1)	84 (76.4)		85 (57.8)	
Smoker			**0.003**		0.244
No	1596 (89.5)	88 (80.0)		136 (92.5)	
Yes	188 (10.5)	22 (20.0)		11 (7.5)	
Steroid use			**0.034**		0.884
No	1680 (94.2)	98 (89.1)		138 (93.9)	
Yes	104 (5.8)	12 (10.9)		9 (6.1)	
Comorbidities					
CHF	30 (1.7)	2 (1.8)	0.914	3 (2.0)	0.747
Hypertension	1202 (67.4)	84 (76.4)	0.052	82 (55.8)	**0.005**
COPD	144 (8.1)	6 (5.5)	0.327	5 (3.4)	**0.049**
Bleeding disorder	58 (3.3)	3 (2.7)	0.763	12 (8.2)	**0.003**
Disseminated cancer	5 (0.3)	0 (0.0)	0.993	1 (0.7)	0.418
Total operation time (minutes)			0.781		**0.005**
0–79	400 (22.4)	25 (22.7)		26 (17.7)	
80–128	720 (40.4)	46 (41.8)		46 (31.3)	
≥ 129	664 (37.2)	39 (35.5)		75 (51.0)	

*WBC* white blood cell; *BMI* body mass index; *ASA* American Society of Anesthesiologists; *CHF* congestive heart failure; *COPD* chronic obstructive pulmonary disease

**Table 3:** Univariate analysis of 30-day postoperative complications in patients with normal WBC count, leukocytosis, and leukopenia. Bold *P* values indicate statistical significance with *P* < .05

Complications	Normal (4500–11,000)	Leukocytosis (≥ 11,000)		Leukopenia (≤ 4500)	
	Number (%)	Number (%)	*P* value	Number (%)	*P* value
Any complication	464 (26.0)	40 (36.4)	**0.018**	42 (28.6)	0.497
Sepsis	9 (0.5)	3 (2.7)	**0.011**	0 (0.0)	0.683
Septic shock	1 (0.1)	0 (0.0)	0.999	0 (0.0)	0.962
Pneumonia	3 (0.2)	0 (0.0)	0.997	2 (1.4)	**0.022**
Unplanned reintubation	4 (0.2)	0 (0.0)	0.999	0 (0.0)	0.999
UTI	12 (0.7)	1 (0.9)	0.772	1 (0.7)	0.991
Cardiac arrest or MI	7 (0.4)	1 (0.9)	0.431	0 (0.0)	0.995
Stroke	0 (0.0)	0 (0.0)	–	0 (0.0)	–
Blood transfusions	58 (3.3)	4 (3.6)	0.826	6 (4.1)	0.590
DVT	12 (0.7)	1 (0.9)	0.772	1 (0.7)	0.991
PE	11 (0.6)	1 (0.9)	0.709	0 (0.0)	0.999
On ventilator > 48 h	2 (0.1)	0 (0.0)	0.986	0 (0.0)	1.000
SSI	40 (2.2)	6 (5.5)	**0.040**	5 (3.4)	0.374
Wound dehiscence	3 (0.2)	1 (0.9)	0.144	0 (0.0)	0.992
Acute renal failure	1 (0.1)	0 (0.0)	0.999	1 (0.7)	0.077
*Clostridioides difficile* infection	1 (0.1)	0 (0.0)	0.999	1 (0.7)	0.077
Non-home discharge	124 (7.0)	14 (12.7)	**0.026**	10 (6.8)	0.946
Readmission	66 (3.7)	10 (9.1)	**0.007**	14 (9.5)	**0.001**
Unplanned reoperation	51 (2.9)	7 (6.4)	**0.044**	7 (4.8)	0.199
Periprosthetic fracture	0 (0.0)	0 (0.0)	–	0 (0.0)	–
LOS > 2 days	329 (18.4)	29 (26.4)	**0.041**	24 (16.3)	0.524
Mortality	2 (0.1)	0 (0.0)	–	0 (0.0)	–

*WBC* white blood cell; *UTI* urinary tract infection; *MI* myocardial infarction; *DVT* deep vein thrombosis; *PE* pulmonary embolism; *SSI* surgical site infection; *LOS* length of stay

**Table 4:** Multivariate analysis, adjusted for all significant and near-significant patient demographics and comorbidities, of 30-day postoperative complications in patients with normal WBC count, leukocytosis, and leukopenia. Bold *P* values indicate statistical significance with *P* < .05

	Leukocytosis (≥ 11,000)	Leukopenia (≤ 4500)
Complications	OR, P value, (95% CI)	OR, P value, (95% CI)
Any complication	1.71, **0.012**, (1.13–2.59)	–
Sepsis	5.31, **0.015**, (1.38–20.37)	–
Pneumonia	–	14.98, **0.004**, (2.32–96.56)
SSI	2.37, 0.060, (0.96–5.82)	–
Non-home discharge	2.18, **0.013**, (1.18–4.05)	–
Readmission	2.76, **0.005**, (1.36–5.63)	2.78, **0.001**, (1.49–5.17)
Unplanned reoperation	2.07, 0.089, (0.89–4.80)	–
LOS > 2 days	1.68, **0.028**, (1.06–2.66)	–

*SSI* surgical site infection; *LOS* length of stay

**Table 5:** Non-home discharge locations and counts of the normal and leukocytosis cohorts

Discharge location	Normal (4500–11,000) Number (%)	Leukocytosis (≥ 11,000)
Skilled care	62 (50.0)	11 (78.6)
Rehab	24 (19.4)	1 (7.1)
Acute care	7 (5.6)	0 (0)
Other facility	31 (25.0)	2 (14.3)

After univariate analysis, leukopenia—versus normal WBC—was associated with lower BMI (*P* = 0.001) and lower rates of hypertension (*P* = 0.005) and COPD (*P* = 0.049), but higher rates of bleeding disorders (*P* = 0.003) and longer total operative time (*P* = 0.005) (Table 2). It was also linked to increased rates of pneumonia (*P* = 0.022) and readmission (*P* = 0.001) (Table 3). Multivariable logistic regression, adjusted for all significant (*P* < 0.05) and near-significant (*P* < 0.2) patient demographics and comorbidities, revealed that patients with baseline leukopenia undergoing aseptic revision TSA had significantly higher adjusted odds of pneumonia (OR 14.98, 95% CI 2.32–96.56; *P* = 0.004) and readmission (OR 2.78, 95% CI 1.49–5.17; *P* = 0.001) compared to those with baseline normal WBC counts (Table 4).

## Discussion

In this study, we analyzed preoperative WBC values and associated postoperative complications in patients undergoing revision TSA. We analyzed 2,041 cases of revision TSA recorded in NSQIP from 2015 through 2022. Patients were grouped into three categories based on leukocyte status: normal, leukocytosis, or leukopenia. After controlling for significant patient demographics and comorbidities, patients in the leukocytosis category were independently associated with increased rates of experiencing any complication, sepsis, non-home discharge, readmission, and LOS > 2 days. Patients in the leukopenia group were independently associated with a higher risk of experiencing pneumonia or readmission.

The incidence of both primary and revision TSA has been increasing steadily over the past decade [2]. In 2017, over 10,000 revision surgeries were performed, and that number is expected to continue to rise [2]. As the prevalence of these operations increases, the necessity for effective methods of preoperative risk assessment increases. The present study evaluated preoperative WBC status as a predictor of postoperative complications following revision TSA. Preoperative WBC status is easily obtained from routine CBCs and can offer valuable insights when evaluated before surgery. Leukocytosis (elevated WBCs) and leukopenia (decreased WBCs) have studied, established medical implications, but their impact in orthopedic surgery, particularly revision TSA, is underexplored in the literature.

Leukocytosis is often an indicator of active infection, though it can also result from other factors such as malignancy, medication use, asplenia, or even stress [16, 17]. In the present study, leukocytosis was significantly associated with smoking, and chronic steroid use. Previous studies have reinforced these findings, showing a significant association between leukocytosis and smoking and chronic steroid use in patients undergoing primary TSA [12]. Smoking and steroid use contribute to leukocytosis by triggering inflammation or altering immune function. Specifically, smoking can cause low-grade inflammation and oxidative stress, leading to elevated leukocyte levels [18, 19]. Additionally, corticosteroids elevate circulating WBC counts by promoting demargination from vessel walls, even as they suppress other immune functions [20]. These findings highlight the multifactorial causes of leukocytosis in patients with comorbidities.

Leukocytosis was independently associated with increased rates of experiencing any complication, sepsis, non-home discharge, readmission, and LOS > 2 days. The elevated incidence of sepsis has been postulated to result from surgical intervention as these procedures cause tissue insult, possibly triggering a systemic inflammatory response [21]. Prior studies in cardiac surgery patients with preoperative leukocytosis have reported increased rates of sepsis and organ failure commonly resulting from the systemic inflammatory response syndrome. [21, 22]. Cardiac surgical patients with preoperative leukocytosis also had an elevated risk of readmission after surgery [23]. Similarly, in primary TSA, preoperative leukocytosis was independently associated with higher rates of sepsis, readmission, and non–home discharge [12]. These consistent findings across different surgical specialties suggest that leukocytosis serves as a reliable marker for increased postoperative complication risk. These associations emphasize the need for thorough preoperative assessment, especially in patients presenting with leukocytosis. Physicians should be mindful of these risks during preoperative planning, as early identification may help guide interventions that reduce complication rates and improve patient outcomes.

In contrast, patients with leukopenia were found to have differing comorbidities and postoperative complications following revision TSA. Patients with preoperative leukopenia were significantly associated with lower BMI and lower rates of comorbid hypertension and COPD when compared to normal WBC status. Likewise, in primary TSA, preoperative leukopenia was linked with lower BMI, and lower rates of hypertension and COPD [12]. Patients with preoperative leukopenia often have lower BMI, which may be linked to malnutrition or chronic illness [24]. They also show lower rates of comorbid hypertension and COPD, potentially due to a lower prevalence of obesity and associated risk factors. These associations indicate that leukopenia may reflect underlying health conditions that contribute to both reduced body mass and poorer nutritional status. These findings demonstrate that preoperative leukopenia not only reflects underlying health conditions but may also influence postoperative outcomes.

Compared to normal WBC count, leukopenia was independently associated with an increased likelihood of experiencing pneumonia and readmission. Typically, leukopenia signifies a weakened immune state and increased susceptibility to infection. As the number of circulating leukocytes decreases, the risk of infections, such as pneumonia, tends to rise, leading to a higher likelihood of complications and subsequent readmission [25]. In previous literature, leukopenia was found to be an independent predictor of ICU admission, as well as in-hospital mortality and the development of postoperative complications in acute surgical patients [26]. The increased risk of patients with leukopenia and the association with postoperative complications supported by the present study and previous literature underscores the importance of closely monitoring and managing patients with leukopenia to prevent adverse outcomes and improve overall recovery.

By identifying risk factors linked to preoperative labs like WBC count, clinicians can better anticipate and mitigate adverse outcomes. Incorporating WBC into preoperative assessment may guide decisions on surgical candidacy and the need for enhanced postoperative monitoring or care.

One of the main limitations is posed by the NSQIP database restricting collection of data to only 30 days post-operation. Any complication occurring after this 30-day period is therefore not represented. In addition, laboratory values are captured from samples drawn up to 30 days before surgery, introducing variability in timing that could influence WBC measurements and their association with outcomes. The specific etiology of laboratory results is also not recorded, so we cannot distinguish whether leukopenia or leukocytosis was chronic or acute. Furthermore, NSQIP includes only limited data on the past medical history of patients, and doesn’t include past medical history of rheumatoid arthritis, HIV, or autoimmune diseases, limiting our ability to analyze subsets of patients with medical comorbidities affecting leukocyte count. NSQIP does not capture certain outcomes—such as in-hospital mortality—so these could not be evaluated. The database also lacks information on potentially relevant variables (e.g., medication dosages, socioeconomic factors), introducing the possibility of unmeasured confounding. Finally, the relatively small number of patients with WBC values outside the normal range limits statistical power for subgroup analyses. Despite these limitations, our study provides evidence for the association between preoperative leukocyte abnormalities and postoperative complications in revision TSA. These insights may inform future risk-assessment models and guide perioperative management strategies for this patient population.

## Conclusion

In this database study, both preoperative leukopenia and leukocytosis were independently associated with select postoperative complications following aseptic revision TSA. Although we applied conventional WBC thresholds to categorize patients, these findings should be viewed as hypothesis-generating. Prospective studies are needed to validate optimal WBC cutoffs and to develop and test a formal risk-stratification model incorporating WBC count alongside other clinical variables. Such a model could ultimately inform preoperative planning and individualized risk assessment to optimize patient outcomes.

## Data Availability

No datasets were generated or analyzed during the current study.
